# Cytogenetic profile and risk of transformation to acute myeloid leukemia (AML) in Indonesian patients with myelodysplastic syndrome (MDS): a pilot study

**DOI:** 10.12688/f1000research.143170.1

**Published:** 2024-03-08

**Authors:** Noorwati Sutandyo, Agus Susanto Kosasih, Resti Mulya Sari, Lyana Setiawan, Ikhwan Rinaldi, Veronika Juanita Maskito, Yuniar Harris Prayitno

**Affiliations:** 1 Faculty of Medicine, University of Indonesia, Jakarta, Indonesia; 2 Department of Hematology and Medical Oncology, Dharmais National Cancer Center Hospital, Jakarta, Indonesia; 3 Department of Integrated Laboratory, Dharmais National Cancer Center Hospital, Jakarta, Indonesia; 4 Hematology and Medical Oncology Division, Department of Internal Medicine, Dr. Cipto Mangunkusumo National Central General Hospital, Jakarta, Indonesia; 5 Medical Research Staff, Department of Hematology and Medical Oncology, Dharmais National Cancer Center Hospital, Jakarta, Indonesia

**Keywords:** Myelodysplastic syndrome, Acute myeloid leukemia, cytogenetic profile, karyotyping

## Abstract

**Background:**

Cytogenetics is a fundamental examination in the course and management of myelodysplastic syndrome (MDS) since it is widely used as a diagnostic and prognostic indicator for the disease. Some cytogenetic profiles are associated with a higher risk of acute myeloid leukemia (AML) transformation. This is the first study to evaluate the cytogenetic profile of Indonesian patients with MDS.

**Methods:**

This prospective cohort study was conducted at the Cancer Center and several other referral hospitals. Patients with primary MDS aged >18 years were included in the study. Clinical examination, peripheral blood smear, and bone marrow aspiration were performed, followed by cytogenetic examination. The results were further categorized into revised international prognostic scoring system (IPSS-R) scores, and cytogenetic profiles were descriptively presented. Patients were followed up for one year to evaluate AML transformation.

**Results:**

A total of 28 MDS patients, aged 66±12 years, were included in this study. The majority of the patients were male (n=17;60.7%), aged 65 years or above (n=19;67.9%), diagnosed with MDS-MLD (n=14;50%), and had an intermediate cytogenetic group (n=4;14.3%). The IPSS-R score was high in 6 (21.4%) patients and very high risk in 3 (10.7%) patients. During one-year follow-up, AML transformation occurred in 3 (10.7%) patients, and 10 (35.7%) patients ceased. Monosomy 7 was observed in 6 (21.4%) patients but in one metaphase each. Deletion of chromosome 5 (del(5)(q31)), del (16)(q21.1), and del (16)(q11.2) were found in a male patient with MDS-EB1.

**Conclusions:**

Monosomy 7 and deletion of chromosome 5 have been identified in Indonesian patients with MDS. MDS-EB has the highest risk of AML transformation.

## Introduction

Myelodysplastic syndrome (MDS) is a heterogeneous group of myeloid clonal disorders caused by ineffective hematopoiesis or failure of blood cell maturation, resulting in peripheral blood cytopenia and bone marrow failure. ^1^ Patients with MDS have increased susceptibility to acute myeloid leukemia (AML). ^2^ Most MDS cases are de novo or primary, while the remaining 10% are secondary, resulting from prior exposure to radiotherapy or chemotherapy for cancer. ^3^

The reported incidence rate of MDS in the general population is 4.5 per 100,000 people per year, with a higher incidence in men than in women (6.2 vs. 3.3 per 100,000 people per year). ^4^ In the United States alone, more than 10.000 new cases are reported annually. Studies have consistently shown that MDS is more commonly diagnosed in patients with advanced age. ^5^ More than 80% of patients with MDS were first diagnosed at the age of 60 years, with a median age of 76 years, and only 6% of patients had disease onset under 50 years of age. ^6^^,^^7^ Based on the Surveillance, Epidemiology, and End Results (SEER) Medicare database, the incidence of MDS is as high as 75 per 100,000 persons in the population older than 65. ^8^ The incidence of MDS is expected to continue to increase because of the higher incidence of secondary MDS, improved awareness of the disease in the general population and clinicians, and more advanced clinical workups. MDS was more frequent in men than women. ^5^ In Asian countries, the incidence of MDS was reported to be 2- to 4-fold lower, and the age of onset was ten years younger than that in Western countries. ^9^

MDS is considered a preleukemic condition, in which 30% of patients progress to AML. ^10^ Age 65 years old and younger, bone marrow blast count >5%, and transfusion dependence were associated with AML transformation. ^11^ Narayanan *et al*. reported that AML transformation occurred in 10% of MDS patients within six months. ^12^ The pathophysiology of MDS and its progression to AML involves cytogenetic, genetic, and epigenetic factors. ^13^

Cytogenetics plays a vital role in the disease course and management and serves as a diagnostic and prognostic indicator in MDS. ^14^ Chromosomal karyotype is included in the Revised International Prognostic Scoring System (IPSS-R) as a fundamental factor for predicting the clinical progression of the disease and guiding the management plan. ^15^ Through the IPSS-R risk group, a clinician can differentiate patients with higher risk, with the treatment goals of delaying AML transformation and improving overall survival. ^16^ The current standard of management for high-risk patients includes hypomethylating agents, decitabine, azacitidine, and allogeneic stem cell transplants. ^17^

Cytogenetic abnormalities were abnormal in >50% of the patients at diagnosis. ^18^ Amplifications, deletions, and translocations were cytogenetic abnormalities found in 50% of primary MDS patients and 80% of secondary MDS patients. ^19^

Currently, there are no published data regarding the cytogenetic profiles of MDS patients in Indonesia. This study aimed to provide a descriptive overview of patients’ demographic and cytogenetic profiles from the National Cancer Center in Indonesia.

## Methods

### Study design

This prospective cohort study was conducted at the Cancer Center Hospital and the other 13 referral hospitals spread around the Jakarta metropolitan area, which sent bone marrow aspirates to the cancer center for leukemia phenotyping from December 2020 to December 2021. Subjects eligible for inclusion in the study were required to be at least 18 years old and exhibiting indications of primary MDS as determined through comprehensive assessments encompassing their medical history, physical examination, peripheral blood smear, and bone marrow cytomorphology. Patients with secondary MDS were excluded from this study.

The study’s sample size estimation employed the formula designed for determining the sample size in studies focusing on population proportions. The significance level (p) was established at 0.05, corresponding to a 95% confidence interval, resulting in the acquisition of the value ∝ = 1.96. The targeted research power was set at 90%, aligning with a value of Zβ = 1.282. The preferred level of absolute precision (d) was specified as 0.1. The proportion of MDS patients who carry cytogenetic abnormalities is 34%.

$$n=\frac{Z\alpha^{2}\mathrm{PQ}}{d^{2}}$$

$$n=\frac{1.96^{2}\times 0.34\times \left(1-0.34\right)}{0.1^{2}}$$

$$n=\frac{3.8416\times 0.34\times 0.66}{0.1^{2}}$$

n=86.2≈86

Due to the decreasing number of visiting patients during the coronavirus disease (COVID-19) surge in 2020, a total sampling approach was adopted. All potential patients were approached by the hematologist in the respective hospital.

The diagnosis of MDS was established according to International Working Group (IWG) guidelines, which require that a patient must have at least two of the following prerequisites: 1) stable cytopenia for ≥6 months unless accompanied by a specific karyotype or bilineage dysplasia, in which case only two months of stable cytopenia are needed; and 2) the exclusion of other potential disorders as a primary reason for dysplasia, cytopenia, or both. Additionally, the diagnosis of MDS also requires ≥1 of 3 MDS-related (decisive) criteria: 1) dysplasia (≥10% in ≥1 of the three major bone marrow lineages), 2) a blast cell count of 5–19%, and 3) a specific MDS-associated karyotype [for example, del(5q), del(20q), +8, or -7/del(7q)]. ^20^

Demographic and clinical data were collected, including age, sex, weight, height, body mass index (BMI), organomegaly (splenomegaly and/or hepatomegaly), lymphadenopathy, peripheral blood smear result, peripheral and medullary blast percentage, subtypes of MDS, cytogenetic group, and the Revised International Prognostic Scoring System (IPSS-R) score classification.

The IPSS-R score was calculated based on the following variables: cytogenetic subgroup, bone marrow blast percentage, hemoglobin level, platelet count, and Absolute Neutrophil Count (ANC), and the results were subsequently classified as very low, low, intermediate, high, or very high. MDS subtypes were established using the World Health Organization (WHO) 2016 classification, which includes MDS with multilineage dysplasia (MDS-MLD), single lineage dysplasia (MDS-SLD), ring sideroblasts (MDS-RS), excess blasts (MDS-EB -1 and 2), isolated del (5q), and unclassifiable (MDS-U). ^21^

To mitigate the risk of potential bias, all bone marrow aspirate (BMA) samples were promptly transported to the clinical pathology laboratory of the Cancer Center Hospital within a four-hour window from the time of sample collection. A minimum of 10 mL of bone marrow aspirate (BMA) specimens was collected from each subject and separated into two tubes with different anticoagulants, that is, ethylenediaminetetraacetic acid (EDTA) for cytomorphology and heparin for cytogenetics. After short-term culture, the cells were harvested and placed on slides for conventional G-banding karyotyping. Karyotyping was subsequently analyzed using the Automated Cell Imaging System CytoVision ^®^ (Leica Biosystems, USA) and reported in accordance with the International Standard for Human Cytogenomic Nomenclature (ISCN 2016).

Patients were then observed for one year through their routine appointments to determine whether they had transformed into AML by monitoring their periodic peripheral blood results. If any blast cells were found in the peripheral blood or the cytopenia deteriorated, a bone marrow puncture was performed to confirm whether there was an increase in the blast count to ≥20% of the total nucleated cells in the bone marrow.

### Data analysis

All collected data were reported descriptively to provide clinical profiles and participants’ cytogenetics using IBM SPSS Statistics version 25 tabulation.

### Ethical statement

This study was approved by the ethical committee of the Cancer Center Hospital on September 14 ^th^, 2020 (No. 0123/KEPK/IX/2020). Written informed consent was taken from all participants subsequent to their agreement to participate in the study.

## Results

**Figure 1.**
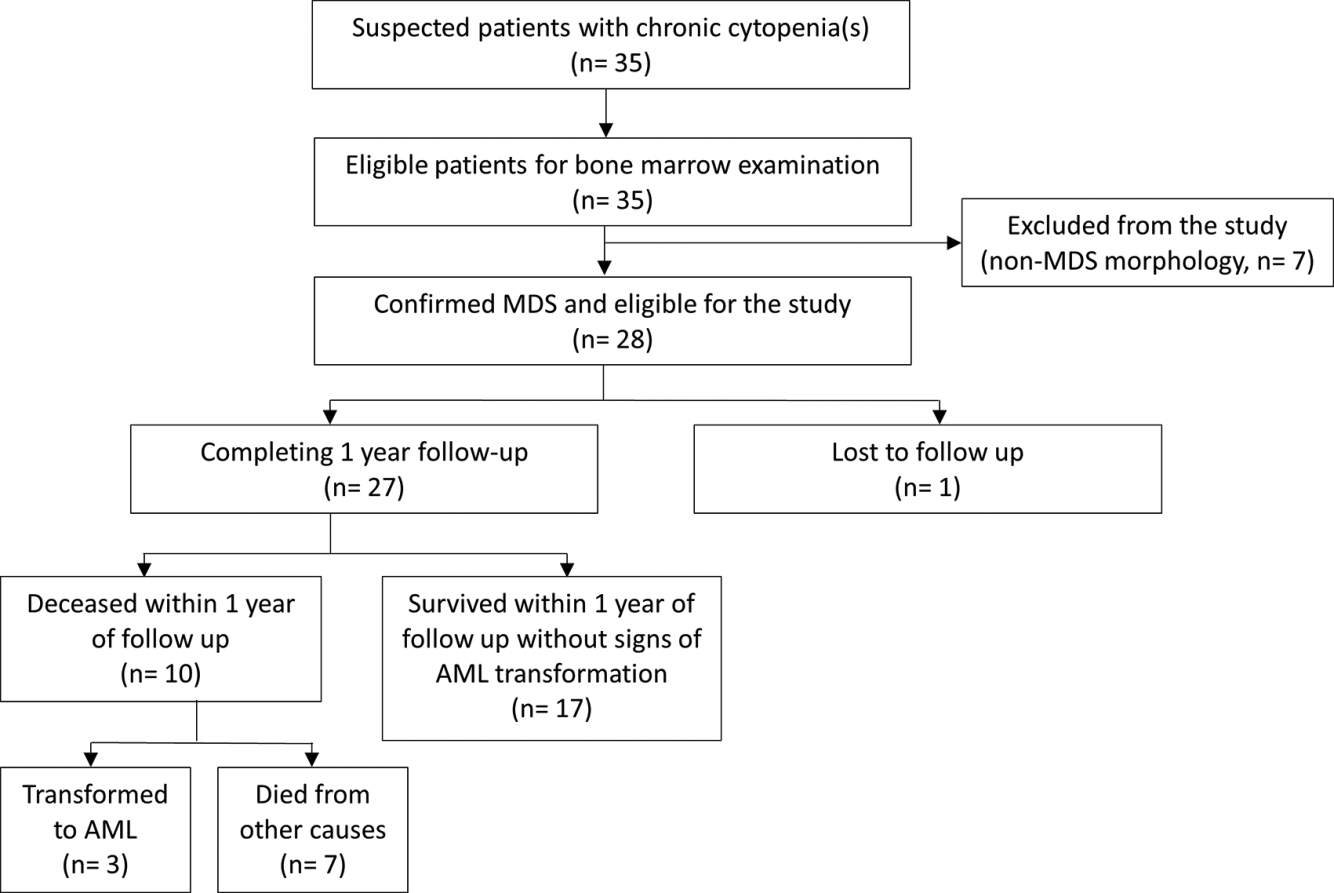
Flow chart of study population.

A total of 28 patients with MDS were included in this study, with a mean age of 66±12 years old. ^41^ We found that nine patients (32.1%) were below 65 years of age, with the youngest being 25 and the oldest being 85. The majority of the participants were male (n=17; 60.7%), and the sex ratio between men and women was 1.54. Six of the nine patients (66.7%) younger than 65 years of age were male. Two (7.14%) patients were aged < 50 years. The most prevalent MDS type in this study population was MDS-MLD (n=14/28; 50.0%), followed by MDS-SLD (n=7/28; 25.0%) and MDS-EB1 (n=5/28; 17.9%). The flow chart of the study population and additional demographic details are shown in Figure 1 and Table 1 respectively.

**Table 1.** Demographic characteristics of the subjects.

Variables		N(%)	Median (min-max)
Age		66.0 ± 12.0	69 (25-85)
	<65	9 (32.1)	
	≥65	19 (67.9)	
Sex			
	Male	17 (60.7)	
	Female	11 (39.3)	
Comorbidity			
	Hypertension	1 (3.6)	
	Diabetes	3 (10.7)	
	Heart disease	1 (3.6)	
	Chronic kidney disease	1 (3.6)	
	None	22 (78.5)	
Hematology			
	Hemoglobin	9.82 ± 2.38	9.15 (6.10-12.30)
	Leukocyte	5.90 ± 3.06	4.35 (0.90-10.0)
	Thrombocyte	167.27 ± 147.58	178.00 (17.00-432.00)
MDS type, n (%)			
	MDS-MLD	14 (50.0)	
	MDS-SLD	7 (25.0)	
	MDS-EB1	5 (17.9)	
	MDS-EB2	2 (7.1)	
Cytogenic group, n (%)			
	Not enough metaphase	19 (67.9)	
	Intermediate	4 (14.3)	
	Poor	2 (7.1)	
	Very poor	3 (10.7)	
IPSS-R score			
	Not applicable	19 (67.9)	
	High	6 (21.4)	
	Very High	3 (10.7)	
AML transformation			
	Yes	3 (10.7)	
	No	25 (89.3)	
Outcome			
	Died	10 (35.7)	
	Alive	18 (64.3)	

MDS, myelodysplastic syndrome; MLD, multilineage dysplasia; SLD, single-lineage dysplasia; EB, excess blasts; IPSS-R, Revised International Prognostic Scoring System; AML. Acute myeloid leukemia

Cytogenetic analysis revealed metaphase in only 14 (50.0%) patients. Due to insufficient metaphase in the sample, cytogenetic grouping could only be done in nine patients (32.1%) ( Table 2). There were 4 patients in the intermediate, 3 in the poor, and 2 in the very poor cytogenetic groups. Based on the IPSS-R category, five patients (17.8%) were at high risk, and three patients (10.7%) were categorized as very high risk, while the remaining 20 patients (71.4%) could not be categorized due to the absence of metaphase during cytogenetic analysis.

**Table 2.** Cytogenetic profiles of the study subjects.

Patients	MDS Type	Cytogenetic profile	Cytogenetic Group	IPSS-R Score	Risk Category	AML Transformation
Male, 59	MDS-EB2	46,XY,-1,+15 [1/10] 41,XY,-8,-9,-11,-12,-18 [1/10] 44,XY,-12,-18 [1/10] 29,XY,+1,+2,+8,+10,+11 [1/10] 26,XY,+1,+2,+5,-10,-11,-18,+19,+20 [1/10] 45,XY,-13 [1/10] 30,XY,+1,+2,+4,+8,+9,+17,+18,-22 [1/10] 46,XY,-2 [1/10] 33,XY,+1,+2,+3,+4,+5,+6,+8,+12,+16,+20,-22 [1/10] 47,XY,+15 [1/10]	Very poor	8	Very high	Yes
Male, 69	MDS-EB1	46,XX,dup(1)(p22p36.3),del(5)(q31),del (16)(q21.1) [8/26]44,idem,-7,add(13(p11.1),-16,-del(16)(q11.2) [18/26]	Very poor	5	High	Yes
Female, 58	MDS-EB1	47 XX, +22 [3/10] 48,XX +13 + 22 [1/10] 55 XX. +3.+14 +16, +17 +19 +19 +20 +21+22 [1/10] 36,X,-4,-5,-6,-8,-9,-10,-12,-16,-17 [1/10] 43,XX, -3,-5,-11,-18, +22 [1/10] 46 X +22 [2/10] 43.X-6-7-8, del (12q), +22 [1/10]	Intermediate	7	Very high	Yes
Male, 67	MDS-SLD	43, XY, -5, -15, -20 [2/15] 44, XY, -18, -19, del(20q) [1/15] 46, XY [12/15]	Poor	5,5	High	No
Male, 81	MDS-MLD	45, XY, -7 [2/10] 45, XY, -16 [1/10] 46, XY [3/10] 47, XY, -7, +13, +22 [3/10] 47, XY, +22 [1/10]	Poor	5,5	High	No
Male, 60	MDS-MLD	23, XY, +1, -7, -11 [1/10] 46, XY, t(1q;3q) [2/10] 46, XY [2/10] 23, XY, t(1q;3q), +1, -2, +3, -7, -11 [1/10] 44, XY, t(1q;3q), -7, -11 [1/10] 46, XY, inv(3q), +8, -18 [1/10] 37, XY, -3, -5, -7, -9, -13, -18, -19, -21, -22 [1/10] 47, XY, -13, +21, +22 [1/10]	Poor	5	High	No
Male, 87	MDS-EB2	32, XY, t(1;12), +1, +3, +10, +12, +13, +16, +17, -18, +19, +21 [1/10] 37, XY, -4, -15, -16, -17, -18, -19, -20, -21, -22, -22, + mar [1/10] 42, XY, -9, -20, -21, -22 [1/10] 44, XY, -7, -22 [1/10] 46, XY [2/10] 46, XY, -9, +13 [2/10] 46, XXY, -9 [1/10] 46, XXY, -9, +13 [1/10]	Intermediate	6,5	Very high	No
Male, 78	MDS-MLD	46, XY [2/7] 45, XY,-2 [2/7] 38,XY,-2,-11,-14,-15,-16,-17,-19,-21 [1/7] 39,XY,-2,-3,-4,-11,-15,-17,-21 [1/7] 42,XY,-12,-14,-15,-21 [1/7]	Intermediate	5,5	High	No
Female, 71	MDS-EB1	46, XX, t(13q; 16q) [3/8] 44,XX,-5, -8 [1/8] 44, XX, t(13q, 16q),-20,-22 [1/8] 38, XX, -2,-5,-6,-7,-11, -12, -13, -18 [1/8] 45, XX, -15, t(13q; 16q) [1/8] 46, XX [1/8]	Intermediate	5,5	High	No
Male, 72	MDS-MLD	42, Y, -X, del(5)(q13, q33), -12, -16, -20 [1/2] 44, Y, -X, +6, +8, -14, -17, -21 [1/2]	NA	NA	NA	No
Male, 60	MDS-MLD	44, XX, -15, -18 [1/3] 45, XX, -15 [2/3]	NA	NA	NA	No
Male, 39	MDS-MLD	24, XY,+1,-8,+12,-14,+15,-22 [1/1]	NA	NA	NA	No
Female, 65	MDS-SLD	45, XX, +8, -19, -22 [1/1]	NA	NA	NA	No
Male, 68	MDS-EB1	41, XY, -7, -11, -12,-14,-15 [1/4] 43,XY,-20,-21,-22 [1/4] 44,XY-7,-11 [1/4] 49,XY,+16,+17.+19 [1/4]	NA	NA	NA	No

MDS, myelodysplastic syndrome; IPSS-R, Revised International Prognostic Scoring System; AML, acute myeloid leukemia acute myeloid leukemia; EB, excess blasts; SLD, single-lineage dysplasia; MLD, multilineage dysplasia.

The cytogenetic profile showed monosomy 7 in six patients. However, it was only found in one metaphase in each patient, so it could not be interpreted as a clonal mutation. Deletion of chromosome 5 (del(5)(q31)), del (16)(q21.1), and del (16)(q11.2) was found in a male patient with MDS-EB1, considered a very poor cytogenetic group, who had AML transformation three months after the diagnosis. The patient died one month after the transformation to AML. In total, three patients developed AML during follow-up.

The 1-year overall survival rate in this study was 64.3%, with 10 of 28 patients deceased. The causes of death were AML (n=3; 30%), COVID-19 (n=3; 30%), bacterial pneumonia (n=2; 20%), cerebrovascular disease (n=1; 10%), and complications of diabetes mellitus (n=1;10%). In the deceased group, the median age of the patients was 69 (55-85) years, with a female-to-male ratio of 1 to 2.6, and the majority of them were MDS-EB1 (40%). The cytogenetic group, which was successfully identified in 9 patients, ranged from high to very high risk. In the surviving group, the median age of the patients was 69 (25-82) years, the female-to-male ratio was 1 to 1.125, and the majority of subjects had MDS-MLD (50.0%). Unfortunately, only three cytogenetic profiles were identified in this group, which indicated a high risk.

## Discussion

In this study, more than half of the patients with MDS were male. This finding was in accordance with the epidemiology of MDS in the general population, which showed a global predominance in males globally. ^22^^,^^23^ The sex ratio within our study is within the reported range, which is 1.24-2.125. ^9^

We found that almost one-third of our study population was diagnosed under 65 years of age, younger than the general population of MDS patients. Based on the Dutch registry, the median age of the patients with MDS at the first diagnosis was 74. ^24^ In Europe, the reported median age at diagnosis is 76 years. ^6^ It has been highlighted that there was a difference in median age between Asian and Western countries, as reported by studies from China and Japan, ranging from 62 to 76 years old. ^25^^,^^26^ The difference in age of onset between Asian and Western countries might be explained by differences in environmental exposure. Genetic factors may also have a significant effect. Less than 10% of the patients were diagnosed under 50 years of age, which was in accordance with a prior study. ^27^

Our data show that the MDS-MLD subtype has the highest frequency among other subtypes, constituting half of the study population. This finding is in accordance with a previous study showing that MDS-MLD was a subtype more represented in Asia, including Japan and China, with 43% and 41.2% of cases, respectively. In contrast, MDS unclassifiable (MDS-U) was ranked as the most prevalent type in Western countries, while the MDS-MLD had a much lower percentage (approximately 7.35%). ^9^ Both Japanese and Chinese studies have reported that MDS-EB is the second most prevalent type of EB. ^28^^–^^30^ However, this was not the case in our study population, in which MDS-SLD was the second most common, followed by MDS-EB-1.

We found that most MDS patients in our study sample had high and very high risks. This finding supports the notion that Asian patients have a higher risk of developing high-risk MDS. Two studies reported a higher frequency of intermediate risk and greater risk in Japanese patients than in more prevalent low-risk patients in Germany and Caucasians. ^30^^,^^31^ Jiang *et al*. also confirmed a similar finding, compiling some Asian and Western studies. The study found that more Asian MDS patients had intermediate-, high-, and very high-risk, while in Western countries, very low- and low-risk patients had a higher distribution. The higher proportion of high-risk cytogenetic abnormalities in Asian patients might explain this finding. ^9^

This study identified one subject with del(5q) and classified it as the MDS-MLD subtype. Del(5q) cytogenetics was associated with a good prognosis and was found to be twice as frequent in Asian patients, with a 4.63% prevalence than in Western countries (8.81%). The most common chromosomal abnormality in MDS is the deletion of the long arm of chromosome 5 (5q), which is found in up to 15% of diagnosed cases. ^32^^,^^33^ del(5q) is more prevalent in Western patients with MDS than in Asian patients. Patients with isolated del(5q) are known to be lenalidomide-responsive, which results in a better prognosis. Anomaly of chromosome 7, either monosomy or deletion of 7q, is reported in approximately 10% of de novo MDS cases and up to 50% of therapy-related MDS cases. Monosomy 7 is correlated with worse prognosis and decreased overall survival in patients with MDS. In addition, there are hundreds of variants of cytogenetic abnormalities that have rarely been reported in MDS patients, such as -X, 3q abnormalities, +13/del(13q), i(17q), +21/-21. In a German study, rare chromosomal abnormalities occurred in less than 2% of patients with MDS. Additionally, we found del(20q) in one patient, although it was not a clonal mutation. Del(20q) is more prevalent in Asian patients than in Western patients, along with trisomy 8.

The underlying cytogenetic discrepancy between Asian and Western countries remains unknown, but it is thought to explain the severity of MDS in Asian patients. Although the incidence of MDS is lower in Asia, very high-risk, high-, and intermediate-risk groups, based on IPSS-R scores, are more prevalent than in Western countries, which reported more MDS patients in very low- and low-risk groups. The MDS-RS, MDS-del(5q), and MDS-U subtypes are more common in Western countries. In contrast, MDS-SLD, MDS-MLD, and MDS-EB are more common in Asian countries.

Several comorbidities have been found to influence disease risk factors, such as congestive heart failure and chronic obstructive pulmonary disease, which are associated with shorter survival. Disease prognosis varies, ranging from indolent to progressive disease. ^34^ The median overall survival in patients with MDS is only 5 years, which is considered to have a poor prognosis.

On average, 30% of patients with MDS develop transformation to AML during the course of the disease. In this study, the rate of AML transformation was 10.7% in one year. This finding is lower than that reported by Vamsi *et al*., who reported that AML transformation during a 1-year follow-up was 26.9% in higher-risk MDS patients, which were patients with intermediate IPSS-R prognostic risk or greater. ^16^ Another study with eight years of follow-up reported AML transformation in 13.9% of MDS patients, with a median of 5 (1-23) months. ^35^ It has been reported that approximately 10-35% of MDS cases evolve into acute leukemia during the disease course. The IPSS-R scoring system was used to estimate the risk of AML transformation five years after diagnosis.

Based on the survival outcome, the overall survival rate in this study population was 64.3%; compared with other studies, the overall 3-year survival rate was 32%. ^36^ Regarding MDS type, MDS-EB1 was reported as the most prevalent diagnosis in the ceased group. This finding supports previous studies reporting that MDS-EB has the worst prognosis among the other types, with a higher risk of AML transformation and shorter median survival, with 16 and 9 months for MDS-EB and MDS-EB2, respectively. ^37^

To the best of our knowledge, this is the first cytogenetic profiling study conducted in Indonesia. In MDS patients, metaphase cytogenetics is used to identify chromosomal abnormalities in approximately 50% of patients. ^32^ However, a limitation of our study is that most of the cytogenic samples had no metaphase, and the cytogenetic profile could only be found in suboptimal amounts (<10 metaphases) of patients (28.57%). It is recommended that when an abnormal result is obtained at diagnosis, at least 20 metaphases should be analyzed. ^38^ Lower metaphase is related to a higher chance of missing small clones. ^39^ Another issue is that we did not identify somatic mutations in the patients and thus could not evaluate the role of somatic mutations.

Considering that karyotyping in MDS therapeutic plans and prognosis has a significant influence, further evaluation is needed throughout the cytogenetic analysis process, from transportation and processing of samples, in which errors could occur at any point. A study from India reported that half of the culture failures occurred in samples processed for 24 hours and above. Technical errors include aged samples, low and high cell counts, the volume of the sample, and the culture technique. ^40^ It should also be noted that cytogenetic testing is not routinely performed in Indonesia.

## Conclusion

MDS is heterogeneous in clinical manifestation and molecular etiology, with AML transformation as one of the final endpoints in the clinical course. Our study observed that MDS-EB was the subtype with the highest risk of AML transformation. Further studies with a larger sample size and modern cytogenetic methods, such as conventional FISH and spectral karyotyping, should be conducted to confirm the findings of this study.

## Data Availability

Figshare: MDS F1000. https://doi.org/10.6084/m9.figshare.25013561.v1. ^41^ This project contains the following underlying data:
• Data MDS.xlsx Data are available under the terms of the Creative Commons Attribution 4.0 International license (CC-BY 4.0).
