# Supplementary material for: Cytogenetic profile and risk of transformation to acute myeloid leukemia (AML) in Indonesian patients with myelodysplastic syndrome (MDS): a pilot study
Source: F1000Res. 2024 Mar 8;13:167. [Version 1] doi: 10.12688/f1000research.143170.1 (PMC11589415; doi:10.12688/f1000research.143170.1)
Supplement: Peer review history

# Reviewer report: Reviewer response for version 1

**DOI:** 10.5256/f1000research.156802.r335617

**Published:** 2024-11-25

**Authors:** Andree Kurniawan

**Affiliations:** 1 Universitas Pelita Harapan, Tangerang, Indonesia

My comments are

 1. The authors have reported cytogenetic profile data in MDS in Indonesia population. This data has high novelty since there was no published data before. considering many difficulty of evaluating cytogenetic in Indonesian because not reimburse yet from national Indonesian Insurance. 

 2. This multi-center study also reported other than cytogenetic profile, potential factors contribute to mortality in MDS in COVID time. 

 3. In the discussion may add potential treatment in regard to finding in cytogenetic profiles.

 4. The idea for Further research after this data should be added at the end of discussion. 

 5. The conclusion may be improve based on the cytogenetic profiles found.

Is the work clearly and accurately presented and does it cite the current literature?

Yes

If applicable, is the statistical analysis and its interpretation appropriate?

Yes

Are all the source data underlying the results available to ensure full reproducibility?

Yes

Is the study design appropriate and is the work technically sound?

Yes

Are the conclusions drawn adequately supported by the results?

Yes

Are sufficient details of methods and analysis provided to allow replication by others?

Yes

Reviewer Expertise:

Supportive care in cancer,  head neck cancer, hematologic malignancies

I confirm that I have read this submission and believe that I have an appropriate level of expertise to confirm that it is of an acceptable scientific standard.

# Reviewer report: Reviewer response for version 1

**DOI:** 10.5256/f1000research.156802.r256195

**Published:** 2024-09-11

**Authors:** William Ying Khee Hwang

**Affiliations:** 1 Duke-NUS Medical School, Singapore, Singapore

This is the first paper reporting the cytogenetic profile of patients with myelodysplastic syndromes in Indonesia. It correlates some of these with clinical outcomes with a one-year follow-up. The sample size is small, but the authors did try to determine the appropriate sample size. The article is written in a clear manner and the conclusions are sound, though the study should probably be expanded to include more patients later. Hopefully, this will encourage routine karyotyping for patients with MDS in Indonesia.

 The authors appropriately cite another paper on the Asian population by Jiang Y et al. It might also be relevant to quote another paper coming out of Indonesia on this subject (albeit a meta-analysis) e.g. [1]

Is the work clearly and accurately presented and does it cite the current literature?

Yes

If applicable, is the statistical analysis and its interpretation appropriate?

Yes

Are all the source data underlying the results available to ensure full reproducibility?

Yes

Is the study design appropriate and is the work technically sound?

Yes

Are the conclusions drawn adequately supported by the results?

Yes

Are sufficient details of methods and analysis provided to allow replication by others?

Yes

Reviewer Expertise:

Haematologic malignancies

I confirm that I have read this submission and believe that I have an appropriate level of expertise to confirm that it is of an acceptable scientific standard.

## References

[1] : Association of Somatic Gene Mutations with Risk of Transformation into Acute Myeloid Leukemia in Patients with Myelodysplastic Syndrome: A Systematic Review and Meta-Analysis. *Asian Pac J Cancer Prev* .2022;23(4) : 10.31557/APJCP.2022.23.4.1107 1107-1116 10.31557/APJCP.2022.23.4.1107 35485665 PMC9375606
